# Speech digital biomarker combined with fluid biomarkers can predict Alzheimer’s type cognitive impairment through machine learning

**DOI:** 10.1002/alz70861_108196

**Published:** 2025-12-23

**Authors:** Gang Wang, Jin‐Tao Wang

**Affiliations:** ^1^ Department of Neurology, Renji Hospital Affiliated to Shanghai Jiaotong University School of Medicine, Shanghai 200217, Shanghai, Shanghai China; ^2^ Department of Neurology, Renji hospital affiliated to Shanghai Jiaotong University School of Medicine, Shanghai, Shanghai China

## Abstract

**Background:**

Current methods for the early detection of Alzheimer's disease (AD) are constrained by high costs, invasiveness, and limited accessibility, underscoring the urgent need for alternative approaches that are accessible, affordable, and patient‐friendly. Previous research has identified speech analysis as a promising tool for the early diagnosis of cognitive impairment (CI). However, only a few small‐size studies have involved the correlation with AD pathology, which the results are not clear. Moreover, monomodal diagnostic tools cannot adequately perform diagnostic tasks. We need to further explore the relationship through large‐sample analysis and further construct models that can diagnose CI or even Aβ status.

**Method:**

1200 participants included AD (*n* =501), amnestic mild cognitive impairment (aMCI) (*n* =461) and cognitively normal controls (NC) (*n* =238) were recruited. The participants underwent neuropsychological tests, speech recordings of the "cookie‐theft" task, serum biomarker quantification, APOE genotyping, and part of them enderwent Aβ PET imaging. Partial Correlation Analysis and LOWESS was used to explore the correlation between speech digital biomarkers and other core AD biomarkers. Finally, machine learning such as XGBoost and Logistic regression were used for constructing the most cost‐effective models for CI and Aβ status prediction, leveraging SHAP values for screening.

**Result:**

Speech digital biomarker PSD correlated with cognitive level, serum serum glial fibrillary acidic protein (GFAP), neurofilament light chain (NFL), phosphorylated tau protein 217 (*p* ‐Tau217) and amyloid deposition. Additionally, A two‐stage change pattern of PSD, GFAP, *p* ‐Tau217 in different Aβ deposition stages was found. Based on the findings, a machine learning CI diagnostic model (AUC=0.924, 95% CI 0.892 ‐ 0.955) incorporating PSD, APOE, GFAP, and demographic info was developed. Furthermore, a robust Aβ status prediction model (AUC=0.888, 95% CI 0.834 ‐ 0.941) with PSD, APOE, *p* ‐Tau217, and demographic info was also constructed.

**Conclusion:**

The combination of speech, blood biomarkers, and machine learning technologies provides valuable opportunities for the development of advanced disease prediction strategies, and we hope that in the era of monoclonal antibody drugs, better prediction strategies can promote large‐scale early screening in the population, thereby improving patient treatment outcomes.

